# *N*-(Fluoren-9-ylmethoxy­carbon­yl)-l-leucine

**DOI:** 10.1107/S1600536808014372

**Published:** 2008-05-17

**Authors:** Kazuhiko Yamada, Daisuke Hashizume, Tadashi Shimizu

**Affiliations:** a National Institute for Materials Science, 3-13 Sakura, Tsukuba 305-0003, Japan; b Advanced Technology Support Division, RIKEN, 2-1 Hirosawa, Wako, Saitama 351-0198, Japan

## Abstract

The title compound [systematic name: fluoren-9-yl *N*-(1-carb­oxy-3-methyl­butyl)carbamate], C_21_H_23_NO_4_, exhibits torsion angles that vary from the typical values found in other Fmoc-protected amino acids, *viz.* the orientations of the fluorene and carboxyl groups [C—O—C—C = 93.8 (2) and N—C—C=O = −23.6 (2)°]. The crystal structure exhibits two inter­molecular hydrogen bonds (O—H⋯O and N—H⋯O) that link the mol­ecules into two-dimensional sheets parallel to the *ab* plane.

## Related literature

For related literature on the structures of *N*-α-Fmoc-protected amino acids, see: Valle *et al.* (1984 ▶); Yamada *et al.* (2008 ▶).
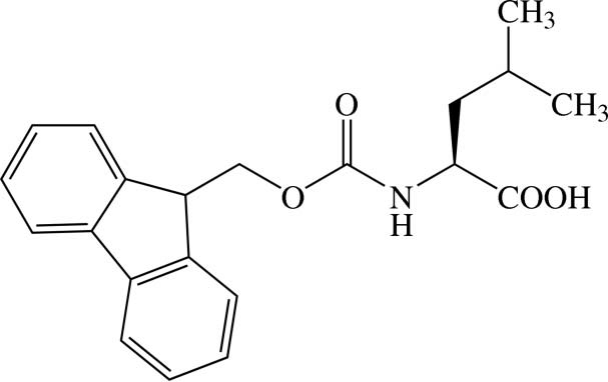


## Experimental

### 

#### Crystal data

C_21_H_23_NO_4_
*M* *_r_* = 353.40
Orthorhombic, 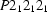

*a* = 5.4953 (1) Å
*b* = 14.2700 (3) Å
*c* = 24.3759 (6) Å
*V* = 1911.51 (7) Å^3^
*Z* = 4
Mo *K*α radiation
μ = 0.09 mm^−1^
*T* = 150 K
0.40 × 0.08 × 0.06 mm

#### Data collection

Rigaku AFC-8 diffractometer with Saturn70 CCD detector
Absorption correction: none
40257 measured reflections
3207 independent reflections
2906 reflections with *I* > 2σ(*I*)
*R*_int_ = 0.055

#### Refinement

*R*[*F* ^2^ > 2σ(*F* ^2^)] = 0.039
*wR*(*F* ^2^) = 0.111
*S* = 1.09
3207 reflections
327 parameters
All H-atom parameters refined
Δρ_max_ = 0.27 e Å^−3^
Δρ_min_ = −0.26 e Å^−3^

### 

Data collection: *CrystalClear* (Rigaku/MSC, 2005 ▶); cell refinement: *HKL-2000* (Otwinowski & Minor, 1997 ▶); data reduction: *HKL-2000*; program(s) used to solve structure: *SIR2004* (Burla *et al.*, 2005 ▶); program(s) used to refine structure: *SHELXL97* (Sheldrick, 2008 ▶); molecular graphics: *ORTEP-3 for Windows* (Farrugia, 1997 ▶); software used to prepare material for publication: *SHELXL97*.

## Supplementary Material

Crystal structure: contains datablocks I, global. DOI: 10.1107/S1600536808014372/fl2198sup1.cif

Structure factors: contains datablocks I. DOI: 10.1107/S1600536808014372/fl2198Isup2.hkl

Additional supplementary materials:  crystallographic information; 3D view; checkCIF report

## Figures and Tables

**Table 1:** Hydrogen-bond geometry (Å, °)

*D*—H⋯*A*	*D*—H	H⋯*A*	*D*⋯*A*	*D*—H⋯*A*
O2—H2*H*⋯O3^i^	0.85 (3)	1.82 (3)	2.6558 (17)	167 (3)
N1—H1*N*⋯O1^ii^	0.87 (3)	2.24 (3)	3.0751 (18)	161 (2)
C8—H8*A*⋯O1^iii^	0.90 (2)	2.51 (2)	3.392 (2)	166 (2)

Symmetry codes: (i) 
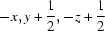
; (ii) 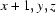; (iii) 
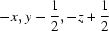
.

**Fig. 1.**
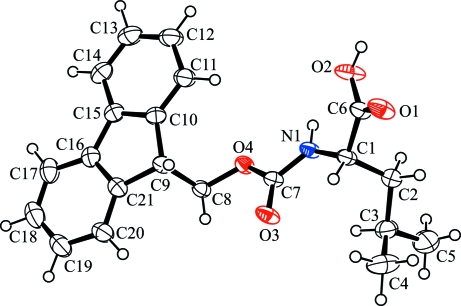
A view of the molecular structure of (I), showing the atom labeling scheme. Displacement ellipsoids are drawn at the 50% probability level.

**Fig. 2.**
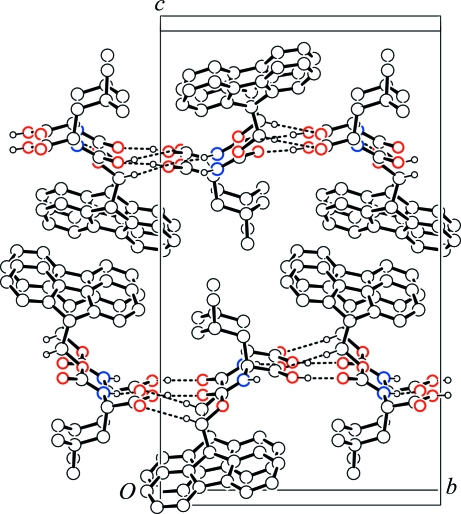
A packing diagram of (I) viewed. The hydrogen atoms were omitted for clarity, except for those forming the hydrogen bonds. Broken lines indicate the hydrogen bonds.

## supplementary crystallographic information

### Comment

The fluoren-9-ylmethoxycarbonyl (Fmoc) group is currently one of the most frequently used protecting groups for peptide synthesis since rapid cleavages can be readily achieved under mild basic conditions with racemization-free results. Almost all the Fmoc-protected twenty-*L*-amino acids are commercially available. The crystal structures of *N*-α-Fmoc-protected-*L*-alanine monohydrate (II, Valle *et al.*, 1984) and *L*-serine (III, Yamada *et al.*, 2008) have been reported so far. In the present study, we have carried out the crystal structure analysis of *N*-α-Fmoc-*L*-leucine, (I).

The bond distances and bond angles of (I, Fig. 1) are consistent with the typical values of Fmoc-protected amino acids found in the other crystal structures. Some torsion angles, however, are found to be quite different. The torsion angle of O2—C6—C1—N1, for example, is -23.6 (2)°, which is in disagreement with the previous observations in the Fmoc-protected amino acids in which the corresponding angles are 150.6° and 175.8° for (II) (Valle *et al.*, 1984) and (III) (Yamada *et al.*, 2008), respectively. Another example is that the torsion angle of C6—C1—N1—C7 in (I) is found to be -134.51 (15)°, which is in reasonable agreement with that of (II), -151.6°, but is inconsistent with that found in (II), -65.6°. Each angle between the fluorene ring and the NC(δb O)O plane is found to be different among the three Fmoc-protected amino acids. The torsion angles C7—O4—C8—C9 and O4—C8—C9—C10 for the title compound, for instance, are 93.78 (16)° and 60.54 (17)°, respectively. On the other hand, the corresponding angles are -179.7° and -172.1°, and 121.9° and -68.2° for (II) and (III), respectively.

Crystals of (I) contain two intermolecular hydrogen bonds (Table 1), which are formed between the carboxyl (O2—H2H) and amide oxygens (O3), and between the amide (N1—H1N) and the carbonyl (O1). The molecules are linked by O2—H2H···O3 hydrogen bonds to form a chain structure along the *b* axis. The linkage is supported by an additional C—H···O interaction (C8—H8A···O1). The chains are joined together by the N1—H1N···O1 hydrogen bonds to form a sheet structure parallel to the *ab* plane. The Fmoc and *i*-butyl moieties are packed between the sheets (Fig. 2).

### Experimental

A powdered sample (I) was obtained from Wako Pure Chemical Industries, Ltd. (Osaka, Japan) and was used for crystallization without further purifications. Colourless needle like crystals of (I) were slowly grown from a saturated dichloromethane solution.

### Refinement

All H atoms were found in difference maps an refined with isotropic thermal parameters.

### Crystal data

**Table:**

C_21_H_23_NO_4_	*F*_000_ = 752
*M**_r_* = 353.40	*D*_x_ = 1.228 Mg m^−^^3^
Orthorhombic, *P*2_1_2_1_2_1_	Mo *K*α radiation λ = 0.71073 Å
Hall symbol: P 2ac 2ab	Cell parameters from 40402 reflections
*a* = 5.49530 (10) Å	θ = 2.2–30.0º
*b* = 14.2700 (3) Å	µ = 0.09 mm^−^^1^
*c* = 24.3759 (6) Å	*T* = 150 K
*V* = 1911.51 (7) Å^3^	Needle, colourless
*Z* = 4	0.40 × 0.08 × 0.06 mm

### Data collection

**Table:**

Rigaku AFC-8 diffractometer with Saturn70 CCD detector	3207 independent reflections
Radiation source: fine-focus rotating anode	2906 reflections with *I* > 2σ(*I*)
Monochromator: confocal	*R*_int_ = 0.055
Detector resolution: 28.5714 pixels mm^-1^	θ_max_ = 30.0º
*T* = 150 K	θ_min_ = 2.2º
ω scans	*h* = −7→7
Absorption correction: none	*k* = −20→20
40257 measured reflections	*l* = −34→34

### Refinement

**Table:**

Refinement on *F*^2^	Secondary atom site location: difference Fourier map
Least-squares matrix: full	Hydrogen site location: difference Fourier map
*R*[*F*^2^ > 2σ(*F*^2^)] = 0.039	All H-atom parameters refined
*wR*(*F*^2^) = 0.111	*w* = 1/[σ^2^(*F*_o_^2^) + (0.0687*P*)^2^ + 0.1717*P*] where *P* = (*F*_o_^2^ + 2*F*_c_^2^)/3
*S* = 1.09	(Δ/σ)_max_ < 0.001
3207 reflections	Δρ_max_ = 0.27 e Å^−^^3^
327 parameters	Δρ_min_ = −0.25 e Å^−^^3^
Primary atom site location: structure-invariant direct methods	Extinction correction: none

### Special details

**Table:**

Experimental. All Friedel pairs were merged, and all f''s of containing atoms were set to zero.
Geometry. All e.s.d.'s (except the e.s.d. in the dihedral angle between two l.s. planes) are estimated using the full covariance matrix. The cell e.s.d.'s are taken into account individually in the estimation of e.s.d.'s in distances, angles and torsion angles; correlations between e.s.d.'s in cell parameters are only used when they are defined by crystal symmetry. An approximate (isotropic) treatment of cell e.s.d.'s is used for estimating e.s.d.'s involving l.s. planes.
Refinement. Refinement of *F*^2^ against ALL reflections. The weighted *R*-factor *wR* and goodness of fit *S* are based on *F*^2^, conventional *R*-factors *R* are based on *F*, with *F* set to zero for negative *F*^2^. The threshold expression of *F*^2^ > σ(*F*^2^) is used only for calculating *R*-factors(gt) *etc*. and is not relevant to the choice of reflections for refinement. *R*-factors based on *F*^2^ are statistically about twice as large as those based on *F*, and *R*- factors based on ALL data will be even larger.

### Fractional atomic coordinates and isotropic or equivalent isotropic displacement parameters (Å^2^)

**Table:**

	*x*	*y*	*z*	*U*_iso_*/*U*_eq_	
O1	−0.3755 (2)	0.42665 (9)	0.27853 (7)	0.0421 (3)	
O2	0.0123 (2)	0.47101 (9)	0.26982 (7)	0.0455 (4)	
H2H	−0.047 (5)	0.526 (2)	0.2667 (11)	0.055 (7)*	
O3	0.1016 (2)	0.15166 (8)	0.23545 (6)	0.0360 (3)	
O4	0.4680 (2)	0.22208 (8)	0.22403 (5)	0.0306 (2)	
N1	0.1792 (2)	0.29706 (9)	0.26957 (6)	0.0284 (3)	
H1N	0.280 (5)	0.3438 (18)	0.2738 (9)	0.044 (6)*	
C1	−0.0651 (3)	0.31083 (10)	0.29096 (7)	0.0287 (3)	
H1	−0.171 (4)	0.2659 (15)	0.2718 (9)	0.035 (5)*	
C2	−0.0783 (4)	0.29621 (13)	0.35339 (8)	0.0406 (4)	
H2A	0.047 (5)	0.3372 (19)	0.3712 (10)	0.048 (7)*	
H2B	−0.225 (6)	0.322 (2)	0.3692 (13)	0.065 (8)*	
C3	−0.0497 (4)	0.19389 (13)	0.37135 (8)	0.0413 (4)	
H3	0.102 (5)	0.1685 (18)	0.3516 (10)	0.048 (7)*	
C4	−0.2695 (5)	0.13470 (17)	0.35502 (14)	0.0601 (7)	
H4A	−0.312 (6)	0.1369 (19)	0.3131 (11)	0.060 (8)*	
H4B	−0.387 (8)	0.163 (3)	0.3755 (15)	0.088 (11)*	
H4C	−0.262 (6)	0.065 (2)	0.3677 (13)	0.072 (9)*	
C5	0.0014 (9)	0.18903 (19)	0.43291 (10)	0.0705 (9)	
H5A	0.163 (8)	0.227 (3)	0.4437 (15)	0.093 (12)*	
H5B	0.017 (6)	0.124 (2)	0.4463 (11)	0.060 (8)*	
H5C	−0.127 (7)	0.223 (2)	0.4509 (13)	0.072 (9)*	
C6	−0.1606 (3)	0.40869 (10)	0.27831 (7)	0.0299 (3)	
C7	0.2373 (3)	0.21843 (10)	0.24241 (6)	0.0261 (3)	
C8	0.5484 (3)	0.14752 (11)	0.18786 (6)	0.0289 (3)	
H8A	0.476 (5)	0.0932 (16)	0.1976 (9)	0.034 (5)*	
H8B	0.721 (5)	0.1412 (15)	0.1960 (9)	0.034 (5)*	
C9	0.5153 (3)	0.17588 (11)	0.12778 (7)	0.0291 (3)	
H9	0.337 (5)	0.1858 (16)	0.1193 (9)	0.043 (6)*	
C10	0.6613 (3)	0.26233 (11)	0.11325 (7)	0.0308 (3)	
C11	0.6335 (4)	0.35445 (12)	0.13131 (8)	0.0385 (4)	
H11	0.504 (5)	0.3707 (16)	0.1573 (9)	0.035 (5)*	
C12	0.8001 (4)	0.42145 (13)	0.11327 (9)	0.0456 (4)	
H12	0.783 (5)	0.4852 (19)	0.1253 (10)	0.054 (7)*	
C13	0.9889 (4)	0.39782 (15)	0.07795 (8)	0.0459 (5)	
H13	1.117 (5)	0.4495 (19)	0.0642 (11)	0.057 (7)*	
C14	1.0166 (4)	0.30626 (15)	0.05954 (8)	0.0408 (4)	
H14	1.148 (5)	0.2884 (16)	0.0338 (10)	0.048 (6)*	
C15	0.8514 (3)	0.23871 (12)	0.07728 (7)	0.0323 (3)	
C16	0.8333 (3)	0.13832 (12)	0.06424 (6)	0.0329 (3)	
C17	0.9738 (4)	0.08170 (16)	0.03029 (8)	0.0437 (4)	
H17	1.114 (5)	0.1107 (17)	0.0111 (10)	0.048 (7)*	
C18	0.9091 (5)	−0.01199 (16)	0.02442 (8)	0.0505 (5)	
H18	1.003 (6)	−0.0545 (19)	0.0001 (11)	0.060 (7)*	
C19	0.7076 (5)	−0.04829 (14)	0.05165 (8)	0.0473 (5)	
H19	0.671 (5)	−0.1136 (19)	0.0461 (10)	0.053 (7)*	
C20	0.5670 (4)	0.00808 (12)	0.08599 (8)	0.0392 (4)	
H20	0.423 (5)	−0.0159 (18)	0.1060 (11)	0.053 (7)*	
C21	0.6305 (3)	0.10141 (11)	0.09189 (6)	0.0317 (3)	

### Atomic displacement parameters (Å^2^)

**Table:**

	*U*^11^	*U*^22^	*U*^33^	*U*^12^	*U*^13^	*U*^23^
O1	0.0236 (6)	0.0258 (5)	0.0769 (9)	0.0009 (4)	0.0034 (6)	−0.0057 (6)
O2	0.0258 (6)	0.0197 (5)	0.0910 (11)	−0.0004 (4)	−0.0005 (7)	0.0087 (6)
O3	0.0236 (5)	0.0220 (5)	0.0623 (7)	−0.0025 (4)	0.0012 (5)	−0.0085 (5)
O4	0.0245 (5)	0.0259 (5)	0.0415 (6)	−0.0027 (4)	0.0051 (5)	−0.0058 (4)
N1	0.0232 (6)	0.0190 (5)	0.0430 (7)	−0.0026 (5)	0.0012 (5)	−0.0035 (5)
C1	0.0246 (7)	0.0194 (6)	0.0423 (8)	−0.0004 (5)	0.0036 (6)	−0.0008 (5)
C2	0.0521 (11)	0.0276 (7)	0.0422 (8)	0.0008 (8)	0.0104 (8)	0.0005 (6)
C3	0.0447 (10)	0.0325 (8)	0.0466 (9)	0.0005 (8)	0.0056 (8)	0.0076 (7)
C4	0.0468 (13)	0.0424 (11)	0.0911 (19)	−0.0093 (10)	0.0005 (13)	0.0225 (12)
C5	0.115 (3)	0.0485 (12)	0.0480 (11)	0.0009 (17)	0.0066 (16)	0.0115 (10)
C6	0.0243 (7)	0.0214 (6)	0.0440 (8)	−0.0003 (5)	0.0012 (6)	−0.0026 (5)
C7	0.0229 (6)	0.0204 (6)	0.0351 (7)	0.0002 (5)	−0.0014 (5)	0.0006 (5)
C8	0.0270 (7)	0.0235 (6)	0.0361 (7)	0.0019 (6)	0.0027 (6)	−0.0013 (5)
C9	0.0249 (7)	0.0254 (7)	0.0370 (7)	−0.0001 (6)	−0.0018 (6)	−0.0007 (5)
C10	0.0282 (8)	0.0287 (7)	0.0353 (7)	−0.0019 (6)	−0.0020 (6)	0.0030 (6)
C11	0.0407 (10)	0.0293 (8)	0.0455 (9)	−0.0012 (7)	0.0029 (8)	0.0011 (6)
C12	0.0529 (12)	0.0301 (8)	0.0537 (10)	−0.0085 (9)	0.0003 (10)	0.0034 (7)
C13	0.0463 (11)	0.0412 (10)	0.0500 (10)	−0.0140 (9)	−0.0004 (9)	0.0089 (8)
C14	0.0346 (9)	0.0490 (10)	0.0389 (8)	−0.0064 (8)	0.0021 (7)	0.0082 (7)
C15	0.0305 (8)	0.0338 (7)	0.0326 (7)	−0.0004 (6)	−0.0026 (6)	0.0037 (6)
C16	0.0325 (8)	0.0364 (8)	0.0296 (6)	0.0047 (7)	−0.0040 (6)	0.0006 (6)
C17	0.0427 (10)	0.0523 (11)	0.0360 (8)	0.0134 (9)	−0.0003 (8)	−0.0033 (7)
C18	0.0625 (14)	0.0489 (11)	0.0401 (9)	0.0206 (11)	−0.0069 (9)	−0.0108 (8)
C19	0.0671 (14)	0.0341 (9)	0.0406 (8)	0.0092 (9)	−0.0122 (10)	−0.0079 (7)
C20	0.0483 (11)	0.0299 (8)	0.0395 (8)	−0.0001 (8)	−0.0082 (8)	−0.0026 (6)
C21	0.0330 (8)	0.0298 (7)	0.0324 (7)	0.0042 (6)	−0.0060 (6)	−0.0021 (6)

### Geometric parameters (Å, °)

**Table:**

O1—C6	1.208 (2)	C8—H8B	0.97 (2)
O2—C6	1.318 (2)	C9—C10	1.514 (2)
O2—H2H	0.85 (3)	C9—C21	1.515 (2)
O3—C7	1.2217 (18)	C9—H9	1.01 (3)
O4—C7	1.3460 (19)	C10—C11	1.395 (2)
O4—C8	1.4506 (18)	C10—C15	1.405 (2)
N1—C7	1.3413 (18)	C11—C12	1.395 (3)
N1—C1	1.453 (2)	C11—H11	0.98 (2)
N1—H1N	0.87 (3)	C12—C13	1.390 (3)
C1—C6	1.523 (2)	C12—H12	0.96 (3)
C1—C2	1.538 (2)	C13—C14	1.390 (3)
C1—H1	0.98 (2)	C13—H13	1.07 (3)
C2—C3	1.532 (3)	C14—C15	1.393 (3)
C2—H2A	1.00 (3)	C14—H14	0.99 (3)
C2—H2B	0.96 (3)	C15—C16	1.471 (2)
C3—C4	1.527 (3)	C16—C17	1.391 (3)
C3—C5	1.528 (3)	C16—C21	1.405 (3)
C3—H3	1.03 (3)	C17—C18	1.391 (3)
C4—H4A	1.05 (3)	C17—H17	0.99 (3)
C4—H4B	0.91 (4)	C18—C19	1.391 (4)
C4—H4C	1.04 (3)	C18—H18	0.99 (3)
C5—H5A	1.08 (4)	C19—C20	1.395 (3)
C5—H5B	0.98 (3)	C19—H19	0.96 (3)
C5—H5C	0.96 (4)	C20—C21	1.384 (2)
C8—C9	1.530 (2)	C20—H20	0.99 (3)
C8—H8A	0.90 (2)		
			
C6—O2—H2H	111.1 (19)	C9—C8—H8B	109.5 (13)
C7—O4—C8	117.44 (12)	H8A—C8—H8B	107 (2)
C7—N1—C1	120.66 (13)	C10—C9—C21	102.41 (13)
C7—N1—H1N	123.1 (16)	C10—C9—C8	112.11 (13)
C1—N1—H1N	116.1 (16)	C21—C9—C8	108.51 (13)
N1—C1—C6	111.69 (13)	C10—C9—H9	110.6 (13)
N1—C1—C2	112.36 (15)	C21—C9—H9	112.6 (13)
C6—C1—C2	107.96 (13)	C8—C9—H9	110.4 (13)
N1—C1—H1	106.7 (13)	C11—C10—C15	120.30 (16)
C6—C1—H1	107.3 (13)	C11—C10—C9	129.51 (16)
C2—C1—H1	110.7 (13)	C15—C10—C9	110.16 (14)
C3—C2—C1	114.03 (14)	C10—C11—C12	118.34 (19)
C3—C2—H2A	111.3 (15)	C10—C11—H11	120.5 (13)
C1—C2—H2A	108.5 (14)	C12—C11—H11	121.1 (13)
C3—C2—H2B	109.2 (18)	C13—C12—C11	121.27 (19)
C1—C2—H2B	112.6 (18)	C13—C12—H12	119.4 (17)
H2A—C2—H2B	100 (2)	C11—C12—H12	119.3 (17)
C4—C3—C5	112.1 (2)	C14—C13—C12	120.65 (18)
C4—C3—C2	111.80 (19)	C14—C13—H13	118.2 (15)
C5—C3—C2	110.04 (18)	C12—C13—H13	121.1 (14)
C4—C3—H3	108.9 (14)	C13—C14—C15	118.62 (19)
C5—C3—H3	107.1 (14)	C13—C14—H14	121.8 (14)
C2—C3—H3	106.6 (15)	C15—C14—H14	119.6 (14)
C3—C4—H4A	114.4 (17)	C14—C15—C10	120.82 (17)
C3—C4—H4B	100 (2)	C14—C15—C16	130.61 (18)
H4A—C4—H4B	111 (3)	C10—C15—C16	108.56 (15)
C3—C4—H4C	115 (2)	C17—C16—C21	120.53 (18)
H4A—C4—H4C	109 (2)	C17—C16—C15	131.08 (19)
H4B—C4—H4C	107 (3)	C21—C16—C15	108.36 (15)
C3—C5—H5A	112 (2)	C16—C17—C18	118.6 (2)
C3—C5—H5B	112.7 (16)	C16—C17—H17	118.0 (14)
H5A—C5—H5B	109 (3)	C18—C17—H17	123.4 (14)
C3—C5—H5C	107 (2)	C17—C18—C19	120.8 (2)
H5A—C5—H5C	104 (3)	C17—C18—H18	121.1 (16)
H5B—C5—H5C	112 (3)	C19—C18—H18	118.0 (16)
O1—C6—O2	124.21 (15)	C18—C19—C20	120.84 (19)
O1—C6—C1	122.02 (15)	C18—C19—H19	117.2 (17)
O2—C6—C1	113.70 (14)	C20—C19—H19	121.9 (17)
O3—C7—N1	125.15 (15)	C21—C20—C19	118.5 (2)
O3—C7—O4	123.98 (14)	C21—C20—H20	118.9 (15)
N1—C7—O4	110.87 (13)	C19—C20—H20	122.6 (15)
O4—C8—C9	110.58 (13)	C20—C21—C16	120.73 (16)
O4—C8—H8A	109.7 (14)	C20—C21—C9	129.00 (17)
C9—C8—H8A	115.2 (14)	C16—C21—C9	110.22 (14)
O4—C8—H8B	104.0 (13)		
			
C7—N1—C1—C6	−134.51 (15)	C13—C14—C15—C10	−0.3 (3)
C7—N1—C1—C2	103.99 (16)	C13—C14—C15—C16	178.99 (18)
N1—C1—C2—C3	−70.9 (2)	C11—C10—C15—C14	0.7 (3)
C6—C1—C2—C3	165.48 (17)	C9—C10—C15—C14	−177.72 (16)
C1—C2—C3—C4	−69.5 (3)	C11—C10—C15—C16	−178.72 (16)
C1—C2—C3—C5	165.2 (2)	C9—C10—C15—C16	2.82 (18)
N1—C1—C6—O1	159.29 (17)	C14—C15—C16—C17	−0.5 (3)
C2—C1—C6—O1	−76.7 (2)	C10—C15—C16—C17	178.86 (18)
N1—C1—C6—O2	−23.6 (2)	C14—C15—C16—C21	−178.76 (18)
C2—C1—C6—O2	100.39 (19)	C10—C15—C16—C21	0.63 (18)
C1—N1—C7—O3	−3.6 (2)	C21—C16—C17—C18	−0.1 (3)
C1—N1—C7—O4	177.08 (13)	C15—C16—C17—C18	−178.11 (18)
C8—O4—C7—O3	8.3 (2)	C16—C17—C18—C19	0.3 (3)
C8—O4—C7—N1	−172.31 (13)	C17—C18—C19—C20	−0.7 (3)
C7—O4—C8—C9	93.78 (16)	C18—C19—C20—C21	0.8 (3)
O4—C8—C9—C10	60.54 (17)	C19—C20—C21—C16	−0.5 (3)
O4—C8—C9—C21	172.91 (13)	C19—C20—C21—C9	−177.57 (17)
C21—C9—C10—C11	176.89 (18)	C17—C16—C21—C20	0.2 (2)
C8—C9—C10—C11	−67.0 (2)	C15—C16—C21—C20	178.61 (16)
C21—C9—C10—C15	−4.84 (17)	C17—C16—C21—C9	177.72 (15)
C8—C9—C10—C15	111.28 (16)	C15—C16—C21—C9	−3.83 (18)
C15—C10—C11—C12	−0.7 (3)	C10—C9—C21—C20	−177.46 (17)
C9—C10—C11—C12	177.40 (17)	C8—C9—C21—C20	63.8 (2)
C10—C11—C12—C13	0.3 (3)	C10—C9—C21—C16	5.24 (17)
C11—C12—C13—C14	0.1 (3)	C8—C9—C21—C16	−113.45 (15)
C12—C13—C14—C15	−0.1 (3)		

### Hydrogen-bond geometry (Å, °)

**Table:**

*D*—H···*A*	*D*—H	H···*A*	*D*···*A*	*D*—H···*A*
O2—H2H···O3^i^	0.85 (3)	1.82 (3)	2.6558 (17)	167 (3)
N1—H1N···O1^ii^	0.87 (3)	2.24 (3)	3.0751 (18)	161 (2)
C8—H8A···O1^iii^	0.90 (2)	2.51 (2)	3.392 (2)	166 (2)

Symmetry codes: (i) −*x*, *y*+1/2, −*z*+1/2; (ii) *x*+1, *y*, *z*; (iii) −*x*, *y*−1/2, −*z*+1/2.
